# The Effect of *Nigella sativa* Supplementation on Cardiometabolic Health in Patients With Metabolic Diseases: A GRADE‐Assessed Systematic Review and Meta‐Analysis

**DOI:** 10.1002/edm2.70207

**Published:** 2026-03-20

**Authors:** Vali Musazadeh, Melika Jahangir, Mahsa Mahmoudinezhad, Amir Hossein Faghfouri, Farshad Teymoori, Ali Arash Anoushirvani, Mohsen Khaleghian

**Affiliations:** ^1^ Student Research Committee, School of Public Health Iran University of Medical Sciences Tehran Iran; ^2^ Department of Nutrition, School of Public Health Iran University of Medical Sciences Tehran Iran; ^3^ Department of Pharmacy Tehran University of Medical Sciences Tehran Iran; ^4^ Student Research Committee Urmia University of Medical Sciences Urmia Iran; ^5^ Department of Nutrition, School of Medicine Urmia University of Medical Sciences Urmia Iran; ^6^ Maternal and Childhood Obesity Research Center Urmia University of Medical Sciences Urmia Iran; ^7^ Department of Hematology and Oncology, School of Medicine, Firoozgar Clinical Research Development Center Iran University of Medical Sciences Tehran Iran; ^8^ Department of General Surgery, School of Medicine, Hazrat‐Rasool General Hospital Iran University of Medical Sciences Tehran Iran

**Keywords:** blood pressure, glycemic indices, *Nigella sativa*, systematic review, update meta‐analysis

## Abstract

**Background:**

Metabolic diseases, including type 2 diabetes and obesity, are primary drivers of cardiovascular disease (CVD) through the exacerbation of intermediate risk factors such as dysglycemia, hypertension, and central adiposity. *Nigella sativa* supplementation has been studied for its potential cardiometabolic benefits, but evidence from clinical trials remains inconsistent. In relation to this matter, a meta‐analysis was undertaken to present a more precise evaluation of the effect of *N. sativa* supplementation on cardiovascular risk factors in patients with metabolic diseases.

**Methods:**

The review was conducted in accordance with PRISMA guidelines. A random‐effects meta‐analysis was performed to calculate weighted mean differences (WMDs) with 95% confidence intervals. Heterogeneity was assessed using the *I* ^2^ statistic, and sensitivity analyses were conducted to examine the robustness of pooled estimates. Primary outcomes included obesity and glycemic indices, while blood pressure outcomes were considered secondary.

**Results:**

Meta‐analysis of 31 trials with 2145 participants revealed significant reductions in weight (WMD: −1.59 kg; 95% CI: −3.03 to −0.15), and BMI (WMD: −0.51 kg/m^2^; 95% CI: −0.85 to −0.18), SBP (WMD = −3.25 mmHg; 95% CI: −4.44, −2.06), and DBP (WMD = −2.75 mmHg; 95% CI: −4.62, −0.89), FBS (WMD = −18.62 mg/dL; 95% CI: −23.30, −13.95), and HbA1c (WMD = −0.56; 95% CI: −0.79, −0.33) following *N. sativa* supplementation. Also, *N. sativa* had no effect on other indices such as WC, HC, WHR, HOMA‐IR, and insulin.

**Conclusion:**

This meta‐analysis suggests that *N. sativa* supplementation is associated with modest improvements in several cardiovascular risk factors. However, no significant effects were observed for measures of insulin resistance or central adiposity, and the findings should be interpreted cautiously given variability in study quality and outcomes. Further well‐designed trials are needed to clarify its clinical relevance.

## Graphical abstract

This figure summarizes the results of a meta‐analysis of 31 clinical trials evaluating the effects of *Nigella sativa* supplementation on cardiovascular risk factors. *Nigella sativa* supplementation was associated with significant reductions in body weight, BMI, systolic and diastolic blood pressure, and glycemic indices. Overall, the findings suggest that *N. sativa* may serve as a beneficial adjunctive supplement in the management of cardiovascular risk factors.

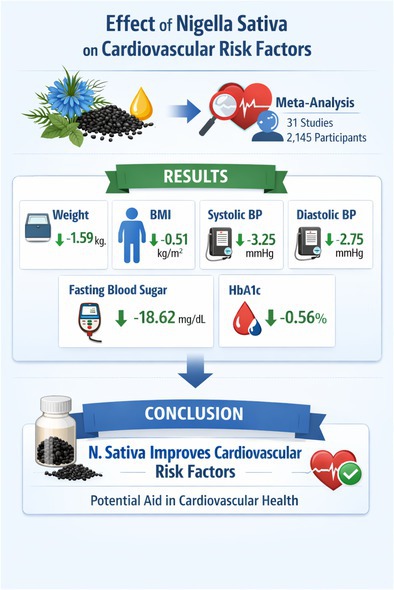

## 1 Introduction

Cardiovascular diseases (CVDs) remain the leading cause of global morbidity and mortality. A primary etiological pathway to CVD is the presence of metabolic diseases, most notably type 2 diabetes mellitus (T2DM) and obesity [1, 2]. These conditions do not operate in isolation; they directly promote atherosclerosis and cardiovascular damage by exacerbating a cluster of intermediate, modifiable cardiovascular risk factors. This cluster includes hypertension, dysglycemia (elevated fasting glucose and HbA1c), insulin resistance, and adverse body composition (e.g., increased central adiposity) [3, 4]. Consequently, the management of metabolic diseases is fundamentally linked to the management of cardiovascular risk, with therapeutic strategies often targeting these same intermediate factors to reduce long‐term CVD events [5, 6]. While several medications for lowering lipids and glucose have been identified, they are associated with a range of adverse side effects [7]. These include renal and hepatic failure, myopathy, gastrointestinal issues, peripheral neuropathy, and rhabdomyolysis [8, 9]. Dietary interventions have been suggested as a beneficial additional treatment to control CVDs [10].

*Nigella sativa* , commonly known as “black seed,” is a medicinal plant belonging to the Ranunculaceae family [11]. It is mainly grown in the Middle East and Southwest Asia. *Nigella sativa* is recognized for its beneficial properties, including anti‐inflammatory, anti‐carcinogenic, antioxidant, and anti‐diabetic effects [12, 13]. However, research on its impact on cardiovascular risk factors has yielded inconsistent findings. The effect of *N. sativa* supplementation on cardiovascular risk factors was assessed by several meta‐analyses [14, 15, 16, 17], although some did not examine changes in outcomes in detail. Therefore, to provide a conclusive and clinically relevant assessment, we conducted this updated systematic review and GRADE‐assessed meta‐analysis. We aimed to precisely quantify the effect of *N. sativa* supplementation on a comprehensive panel of cardiovascular risk factors, including BMI, body weight (BW), waist circumference (WC), hip circumference (HC), and waist‐to‐hip ratio (WHR), SBP, DBP, FBS, insulin, HbA1c, and HOMA‐IR in populations with underlying metabolic diseases. This approach allows for a direct evaluation of its potential utility within the established paradigm of cardiovascular risk factor management.

## 2 Methods

The Preferred Reporting Items for Systematic Reviews and Meta‐Analyses (PRISMA) guideline was adhered to in the preparation of the present systematic review and meta‐analysis [18]. This study's protocol has undergone approval and submission to the prospective register of systematic reviews database (PROSPERO) with the registration number (CRD42023444753).

### 2.1 Search Strategy

A literature search was conducted across six databases, including PubMed (via Medline), Web of Science, Embase, Cochrane Central, and grey literature, to identify studies investigating the impact of *N. sativa* on cardiovascular risk factors until July 2024. No restrictions were imposed on the publication year or language of the studies, and all relevant studies pertaining to the central question were considered. The keywords were defined using Medical Subject Headings (MeSH) and were tailored for each database and are presented in Table S1. Additionally, a manual search of the reference lists of eligible articles was conducted to ensure inclusion of all relevant publications.

### 2.2 Eligibility Criteria

Based on PICO, inclusion criteria include Population: Individuals diagnosed with metabolic diseases, Intervention: *N. sativa* supplementation, Comparison: Placebo or control, Outcome: Impact on weight, BMI, FBS, HbA1c, HOMA‐IR, insulin, SBP, and DBP. The exclusion criteria consisted of the following: (1) Research on conditions unrelated to metabolic diseases; (2) Studies with inadequate data; (3) Simultaneous studies or those with participant overlap; (4) Non‐experimental studies including observational studies, case series, case reports, reviews, conference papers, editorials, and animal experiments; (6) studies adopting qualitative research designs. Two investigators identified studies that met the specified criteria and resolved discrepancies by consulting a third investigator or reaching a consensus.

### 2.3 Study Selection

Two investigators reviewed research studies to determine their eligibility for inclusion. They followed established criteria to conduct a thorough assessment of the text and decided which studies to include or exclude. We obtained the full texts of the studies selected for the analysis. The senior researcher resolved any discrepancies regarding the study designs or methodologies and made the final decisions about which studies to include or exclude.

### 2.4 Data Extraction

VM and AHF independently acquired data from integrated articles. The collected characteristics of the included studies contained the original author, country, study design, population size (both total and detailed), intervention details, control variables, and results. Disputes were resolved through discussions (FSH).

### 2.5 Risk of Bias Assessment

Two independent reviewers assessed the quality of the study using the Cochrane Collaboration modified risk of bias tool [19]. This tool evaluates seven categories of bias: attrition bias, performance bias, reporting bias, allocation concealment, random sequence generation, and other possible causes of bias. Each category of study bias was then categorized as “low,” “high,” or “unclear.”

### 2.6 Quantitative Analysis

Examining the *N. sativa* and control sets, we determined the average alterations and standard deviation (SD) of cardiovascular risk factors. These values derived the overall effect magnitude (weighted mean difference [WMD]). SD was also computed using the standard error (SE) and 95% confidence interval (CI), following the procedure outlined by Hozo et al. [20]. Utilizing a random‐effects model, we combined the unstandardized difference in means. The chi‐squared test and *I* ^2^ were used for assessing the heterogeneity among the studies. Egger's and Begg's tests were applied to evaluate the risk of publication bias. A funnel plot was used to visually represent publication bias. A symmetrical funnel‐shaped distribution indicates a low risk of publication bias, while an asymmetrical distribution suggests a high risk. Additionally, a sensitivity analysis was conducted to assess the robustness of the pooled effect size. All analyses were conducted using Stata software‐16 (STATA Corp, College Station, TX, USA), and statistical significance was considered for *p*‐values below 0.05.

### 2.7 Certainty Assessment

The GRADE standards working group (gradeworkinggroup.org) assessed the overall certainty of evidence from multiple studies. This assessment evaluated evidence quality across four categories: high, moderate, low, and very low [21].

## 3 Results

### 3.1 Study Selection and Characteristics of the Studies

**FIGURE 1:**
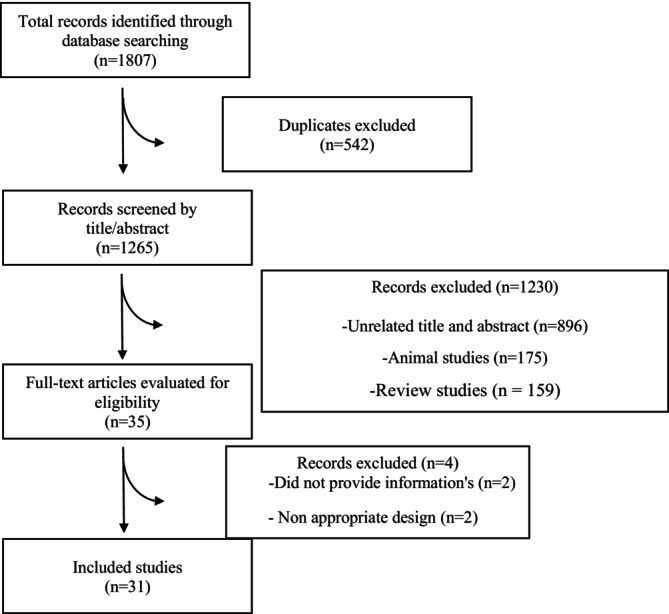
Flow diagram of study selection.

The database searches yielded 1807 records, of which 1265 remained after removing duplicates. Following the first evaluation step, 1230 records were removed, and 35 records remained for full‐text screening. In the second step of screening, four articles were excluded for reasons presented in Figure 1. Finally, 31 studies were included in the meta‐analysis (Figure 1).

Table 1 provides a summary of the characteristics of the included studies. A total of 2145 participants were included, and the publications ranged from 2008 to 2022. The duration of the intervention varied between 4 and 24 weeks, and the sample size ranged from 38 to 144 participants.

**TABLE 1:** Study characteristics of included studies.

Author, year	Design	Participants, *n*	Health condition	Age, year	Intervention		Duration (week)
					Treatment group	Control group	
Dehkordi et al. 2008	RA/dB/parallel	M: 108 Int1: 36, Int2: 39 Con: 33	Mild Hypertension	Int: 44.6, Con: 43.1 Int: 43.7, Con: 43.1	200 mg/day *Nigella sativa* (capsule) 400 mg/day *Nigella sativa* (capsule)	Placebo: Placebo	8
Qidwai et al. 2009	RA/dB/parallel	M/F: 73 Int: 39, Con: 34	Hypercholesterolemia	Int: 45.58, Con: 46.86	1000 mg/day *Nigella sativa* (powder)	Calcium lactate	6
Datau et al. 2010	RA/dB/parallel	M: 39 Int: 19, Con: 20	Obese	30–45	1500 mg/day *Nigella sativa* (capsule)	Flour	12
Shah et al. 2012	RA/parallel	M/F: 159 Int: 81, Con: 78	Metabolic Syndrome	20–65	500 mg/day *Nigella sativa* (capsule) + simvastatin, metformin, enalapril atenolol, clopidogrel	Simvastatin, metformin, enalapril atenolol, clopidogrel	6
Sabzghabaee et al. 2012	RA/parallel	M/F: 74 Int: 37, Con: 37	Hyperlipidemia	Int: 40.38, Con: 38.4	2000 mg/day *Nigella Sativa* (capsule)	Placebo	4
Najmi et al. 2012	RA/parallel	M/F: 80 Int: 40, Con: 40	Metabolic syndrome	20–70	500 mg/day *Nigella sativa* Powder (capsule) + metformin + atorvastatin	Metformin + atorvastatin	8
Hosseini et al. 2013	RA/dB/parallel	M/F: 70 Int: 35, Con: 35	T2DM	Int: 48.74, Con: 50.72	5000 mg/day *Nigella sativa* (oil)	Mineral Oil	12
Amin et al. 2015	RA/dB/parallel	M: 125 Int: 62, Con: 63	Metabolic syndrome	Int: 45.1, Con: 41.57	1500 mg/day *Nigella sativa* (capsule)	Ispaghula capsule	8
Heshmati et al. 2015	RA/dB/parallel	M/F: 72 Int: 36, Con: 36	T2DM	Int: 45.3, Con: 47.5	3000 mg/day *Nigella sativa* oil (soft gel)	Sunflower soft gel	12
Shavakhi et al. 2015	RA/dB/parallel	M/F: 81 Int: 40, Con: 41	Nonalcoholic steatohepatitis	Int: 38.6, Con: 38.5	75 mg/day cumin capsule	Placebo	24
Kaatabi et al. 2015	RA/SB/parallel	M/F: 103 Int: 51, Con: 52	T2DM	Int: 46.82, Con: 46.12	2000 mg/day *Nigella sativa* powder (capsule)	Charcoal	48
Mahdavi et al. 2016	RA/dB/parallel	F: 50 Int: 25, Con: 25	Obese	Int: 41.01, Con: 39.5	3000 mg/day *Nigella sativa* (oil) + low calorie diet	Sunflower oil	8
Hozoori et al. 2016	RA/dB/parallel	M: 67 Int: 37, Con: 30	Overweight	Int: 31.6, Con: 32.1	2500 mg/day *Nigella sativa*	Paraffin oil	8
Rachman et al. 2017	RA/SB/parallel	M/F: 66 Int: 33, Con: 33	Metabolic syndrome risk	> 18	1500 mg/day *Nigella sativa* oil (capsule), 3000 mg/day *Nigella sativa* oil (capsule)	Placebo	3
Mohammadshahi et al. 2018	RA/dB/parallel	M/F: 44 Int: 22, Con: 22	NAFLD	Int: 39, Con: 42.22	1000 mg/day *Nigella sativa* (capsule)	Paraffin oil	8
Rashidmayvan et al. 2019	RA/dB/parallel	M/F: 44 Int: 22, Con: 22	NAFLD	Int: 39, Con: 42.22	1000 mg/day *Nigella sativa* oil (capsule)	Paraffin oil	8
Safi et al. 2020	RA/dB/crossover	F: 39 Int: 19, Con: 20	Overweight and Obese	Int: 38.3, Con: 33.55	2000 mg/day *Nigella sativa* (capsule)	Placebo: paraffin oil	8
Hussain et al. 2017	RA/parallel	M/F: 70 Int: 35, Con: 35	NAFLD	Int: 38, Con: 36	2000 mg/day *Nigella sativa* (capsule)	Placebo: micro crystalline cellulose	12
Khonche et al. 2019	RA/dB/parallel	M/F: 120 Int: 60, Con: 60	NAFLD	Int: 47.9, Con: 45.9	5 mL/day *Nigella sativa* (oil)	Placebo: placebo	12
Razmpoosh et al. 2020	RA/dB/crossover	F: 39	Obese and overweight	Int: 38, Con: 34	2000 mg/day *Nigella sativa* (capsule)	Paraffin oil	8
Rizka et al. 2018	RA/parallel	M/F: 76 Int: 38, Con: 38	HTN in elderly	Int: 72, Con: 73.8	0.6 g/day NS seed extract (capsule)	Placebo: placebo	3
Mohtashami et al. 2019		M/F: 51 Int: 27, Con: 24	Mets	47.5	3 g/day NS powder	Placebo: Placebo	8
Darand et al. 2019	RA/dB/parallel	M/F: 43 Int: 22, Con: 21	NAFLD	Int: 48.9 Con: 46.2	2000 mg/day *Nigella Sativa* (capsule) + lifestyle modification	Rice starch + lifestyle modification	12
Naeimi et al. 2019	RA/dB/parallel	F: 55 Int: 32, Con: 23	PCOS	24	1000 mg/day *Nigella sativa* oil (soft gel)	Sunflower oil	16
Kooshki et al. 2020	RA/dB/parallel	M/F: 50 Int: 25, Con: 25	T2DM	Int: 52.3 Con: 55.91	1000 mg/day *Nigella sativa* oil (capsule)	Medium‐chain triglyceride oils	8
Hadi, Iran, 2020	RA/dB/parallel	M/F: 42 Int: 23, Con: 19	T2DM	Int: 51.4, Con: 56	1000 mg/day *Nigella sativa* (soft gel capsule)	Sunflower oil	8
Shirazi, Iran, 2020	RA/dB/parallel	F: 140 Int: 70, Con: 70	Postmenopausal with metabolic syndrome	Int: 50.6, Con: 50.5	500 mg/day *Nigella sativa* (Capsule)	Placebo	8
Shoaei‐Hagh, Iran, 2021	RA/dB/parallel	M/F: 55 Int: 26, Con: 29	Hypertensive patients	Int: 58.04, Con: 59.92	2500 mg/day *Nigella sativa* (oil)	Sunflower oil	8
Shishehbor, Iran, 2021	RA/parallel	M/F: 38 Int: 20, Con: 18	Hyperlipidemic patients	Int: 39.11, Con: 42.8	90,000 mg/day black seed raisin	Routine nutritional recommendations	5
Mostafa, Egypt, 2021	RA/parallel	M/F: 70 Int: 35, Con: 35	Obese prediabetic subjects	Int: 44.26, Con: 44.85	900 mg/day *Nigella sativa* oil (soft gel capsule)	Lifestyle modification	24
Rahmani, Iran, 2022	RA/dB/parallel	M/F: 41 Int: 20, Con: 21	Diabetic hemodialysis patients	Int: 49.6, Con: 48.57	2000 mg/day *Nigella sativa* oil (soft gel capsule)	Paraffin oil	12

### 3.2 Risk of Bias and Grade Assessment

Table 2 provides the results of the quality assessments of the included studies. 12 out of 31 studies were high quality. Overall risk of bias was low for randomization and attrition, while unclear allocation concealment and reporting bias were the most common concerns. Blinding was generally adequate but often insufficiently reported. High‐quality evidence supported significant effects on weight, BMI, FBS, SBP, and DBP, while very low‐quality evidence was found for WC, HOMA‐IR, and insulin (Table 3). Evidence was downgraded to very low certainty for WC, HOMA‐IR, and insulin primarily due to serious inconsistency across studies, indirectness, and imprecision, reflected by wide confidence intervals and non‐significant pooled effects.

**TABLE 2:** Results of risk of bias assessment.

Study	Random sequence generation	Allocation concealment	Reporting bias	Other sources of bias	Performance bias	Detection bias	Attrition bias
Dehkordi et al. 2008	L	U	L	H	L	L	L
Qidwai et al. 2009	L	L	H	H	L	L	L
Datau et al. 2010	L	U	H	H	L	L	L
Shah et al. 2012	L	U	L	H	U	U	U
Sabzghabaee et al. 2012	L	U	H	H	U	U	L
Najmi et al. 2013	L	U	H	H	U	U	U
Hosseini et al. 2013	L	L	H	H	L	L	H
Amin et al. 2015	L	L	H	H	L	L	L
Heshmati et al. 2015	L	L	L	L	L	L	L
Shavakhi et al. 2015	L	L	H	H	L	L	L
Kaatabi et al. 2015	L	L	L	H	L	H	L
Mahdavi et al. 2016	L	L	L	L	L	L	L
Hozoori et al. 2016	L	L	H	H	L	L	L
Rachman et al. 2017	L	L	H	H	L	H	H
Mohammadshahi et al. 2018	L	U	H	H	L	L	H
Rashidmayvan et al. 2019	L	U	H	H	L	L	H
Safi et al. 2020	L	L	L	L	L	L	L
Hussain et al. 2017	L	L	L	H	U	U	H
Khonche et al. 2019	L	L	H	H	L	L	L
Razmpoosh et al. 2020	L	L	L	L	L	L	L
Rizka et al. 2018	L	L	L	U	L	L	L
Mohtashami et al. 2019	L	U	H	H	L	L	L
Darand et al. 2019	L	L	L	L	L	L	L
Naeimi et al. 2019	L	L	L	L	L	L	L
Kooshki et al. 2020	L	L	H	L	L	L	H
Hadi et al. 2020	L	L	L	L	L	L	L
Shirazi et al. 2020	L	L	H	H	L	L	L
Shoaei‐Hagh et al. 2021	L	L	H	L	L	L	L
Shishehbor et al. 2021	L	U	H	H	U	U	L
Mostafa et al. 2021	L	L	L	H	U	U	L
Rahmani et al. 2022	L	L	L	L	L	L	L

**TABLE 3:** Summary of quality of evidence.

Outcome measures	Summary of findings		Quality of evidence assessment (GRADE)					
	No of patients (trials)	WMD (95% CI)	Risk of bias	Inconsistency	Indirectness	Imprecision	Publication bias	Quality of evidence
Weight	831 (11)	−2.16 (−3.04, −1.29)	Not serious	Not serious	Not serious	Not serious	Not serious	High
BMI	953 (13)	−0.51 (−0.85, −0.18)	Not serious	Not serious	Not serious	Not serious	Not serious	High
WC	696 (10)	−2.76 (−7.20, 1.69)	Not serious	Serious	Serious	Serious	Not serious	Very low
FBS	1519 (22)	−18.62 (−23.30, −13.95)	Not serious	Not serious	Not serious	Not serious	Not serious	High
HbA1c	604 (9)	−0.56 (−0.79, −0.33)	Not serious	Serious	Serious	Not Serious	Not serious	Low
HOMA‐IR	385 (6)	−0.33 (−0.71, 0.05)	Not serious	Serious	Serious	Serious	Not serious	Very low
insulin	452 (9)	0.75 (−1.34, 2.84)	Not Serious	Serious	Serious	Serious	Not serious	Very low
SBP	834 (13)	−3.25 (−4.44, −2.06)	Not serious	Not serious	Not serious	Not serious	Not serious	High
DBP	834 (13)	−2.75 (−4.62, −0.89)	Not serious	Not serious	Not serious	Not serious	Not serious	High

### 3.3 Effects of *N. sativa* Supplementation on Primary Outcomes

#### 3.3.1 Effect of *N. sativa* Supplementation on Obesity Indices

**FIGURE 2:**
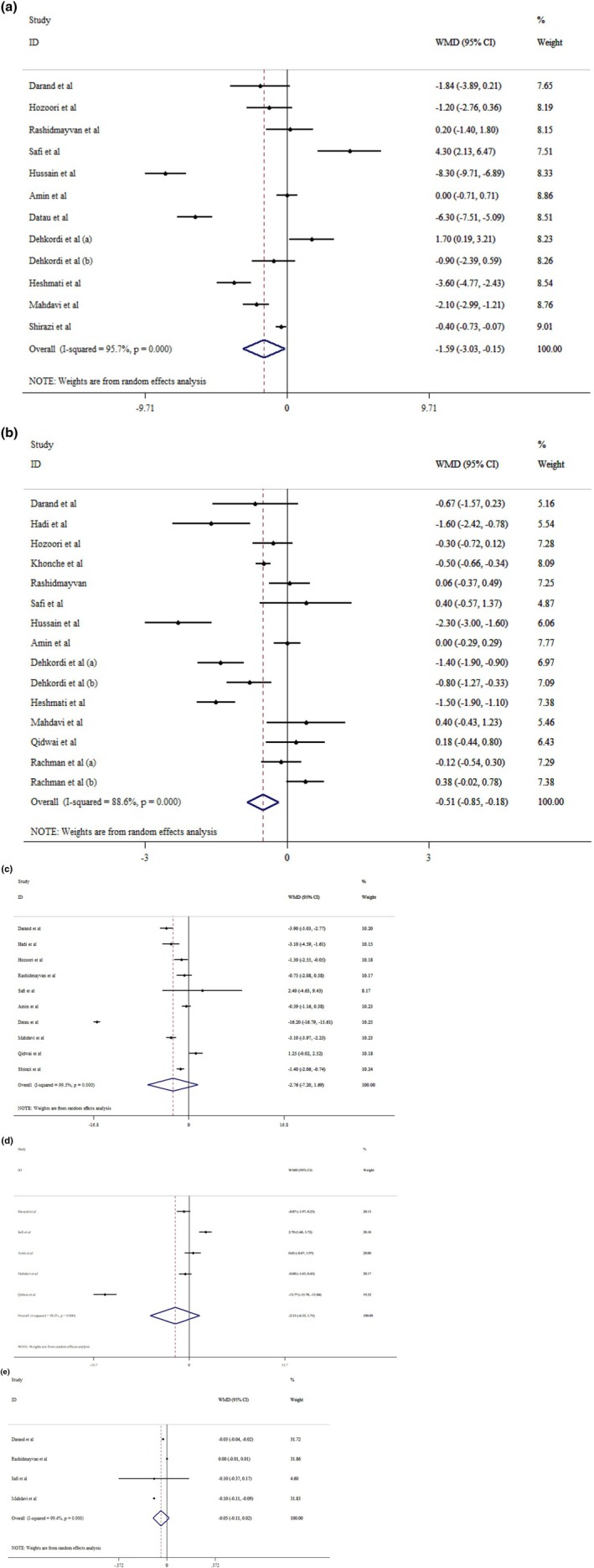
Forest plot detailing mean difference and 95% confidence intervals (CIs) the effects of *Nigella sativa* supplementation on weight (a), BMI (b), WC (c), HC (d), and WHR (e) levels.

Our finding revealed that *N. sativa* could reduce weight (WMD: −1.59 kg; 95% CI: −3.03 to −0.15, *p* = 0.030; *I* ^2^ = 95.7%, *p* < 0.001) (Figure 2a), and BMI (WMD: −0.51 kg/m^2^; 95% CI: −0.85 to −0.18, *p* < 0.001; *I* ^2^ = 88.6%, *p* < 0.001) (Figure 2b), significantly. While it could not have a significant impact on WC (WMD: −2.76 cm; 95% CI: −7.20 to 1.69, *p* = 0.224; *I* ^2^ = 99.5%, *p* < 0.001) (Figure 2c), HC (WMD: −2.31 cm; 95% CI: −6.33 to 1.71, *p* = 0.261; *I* ^2^ = 98.2%, *p* < 0.001) (Figure 2d), and waist‐to‐height ratio (WHtR) (WMD: −0.05; 95% CI: −0.11 to 0.02, *p* = 0.153; *I* ^2^ = 99.4%, *p* < 0.001) (Figure 2e). Subgroup analysis indicated that long‐term supplementation with *N. sativa* could have beneficial effects on weight and BMI levels in patients with T2DM. Specifically, the oil form was associated with weight reduction, while the capsule form showed a positive effect on BMI. In addition, patients with BMI < 30 could benefit more from *N. sativa* supplementation (Table 4). The sensitivity analysis showed that results of the WC and WHtR changed significantly by removing Datau et al. [22], and Rashidmayvan et al. [23], respectively. Egger and Begg test showed a non‐significant small study effect for weight and BMI values (*p* > 0.05). Visually inspection of the funnel plot showed a little asymmetry and we performed a trim‐and‐fill analysis due to publication bias (Figures S1 and S2). Thus, the results after adding three imputed studies for weight still showed a significant change (WMD: −2.71 kg; 95% CI: −4.23 to −1.21, *p* < 0.05).

**TABLE 4:** Subgroup analyses for the effects of *Nigella sativa* supplementation on cardiovascular risk factors.

	No	WMD (95% CI)	*I* ^2^ (%)	*p*‐heterogeneity
**Nigella sativa supplementation on FBS**				
Overall	23	−18.62 (−23.30, −13.95)	99.2	< 0.001
*Age (year)*				
≤ 50	19	−14.93 (−19.78, −10.08)	99.2	< 0.001
> 50	4	−36.64 (−61.61, −11.68)	98.4	< 0.001
*Intervention duration (week)*				
< 12	14	−21.51 (−26.19, −16.83)	98.0	< 0.001
≥ 12	9	−12.80 (−22.77, −2.82)	99.6	< 0.001
*Type of intervention*				
Capsule	11	−10.77 (−14.62, −6.92)	96.8	< 0.001
Oil	12	−27.08 (−35.77, −18.38)	99.5	< 0.001
*Study population*				
Metabolic syndrome	4	−34.39 (−52.88, −15.90)	94.5	< 0.001
T2DM	7	−36.62 (−46.91, −26.33)	97.2	< 0.001
NAFLD	4	−5.70 (−6.53, −4.87)	13.1	0.327
Obese and overweight	3	−0.86 (−2.60, 0.88)	88.0	< 0.001
PCOS	2	−11.91 (−33.37, 9.55)	98.9	< 0.001
Hyperlipidemia	2	−2.27 (−3.25, −1.28)	0.0	0.894
HTN	1	−10.50 (−15.57, −5.43)	—	—
*Sample size*				
≤ 60	11	−20.54 (−29.16, −11.93)	99.0	< 0.001
> 60	12	−15.32 (−19.76, −10.88)	97.9	< 0.001
*BMI*				
≤ 30	14	−21.56 (−28.60, −14.52)	99.3	< 0.001
> 30	6	−12.60 (−21.07, −4.13)	98.3	< 0.001
NR	3	−21.38 (−43.13, 0.37)	99.2	< 0.001
**Nigella sativa supplementation on weight**				
Overall	12	−2.16 (−3.04, −1.29)	95.7	< 0.001
*Intervention duration (week)*				
< 12	8	−0.04 (−0.91, 0.84)	83.6	< 0.001
≥ 12	4	−5.07 (−7.60, −2.55)	92.3	< 0.001
*Type of intervention*				
Capsule	4	−1.52 (−3.57, 0.53)	97.0	< 0.001
Oil	8	−1.76 (−3.20, −0.33)	80.6	< 0.001
*Study population*				
NAFLD	3	−3.33 (−8.87, 2.20)	97.0	< 0.001
Obese and Overweight	4	−1.42 (−4.86, 2.02)	96.2	< 0.001
Metabolic Syndrome	2	−0.33 (−0.63, −0.02)	0.0	0.319
HTN	2	0.40 (−2.15, 2.94)	82.6	< 0.001
T2DM	1	−3.60 (−4.77, −2.43)	—	—
*Sample size*				
≤ 60	4	−0.96 (−5.51, 3.60)	96.6	< 0.001
> 60	8	−1.83 (−3.36, −0.30)	95.6	< 0.001
*BMI*				
≤ 30	8	−1.73 (−4.04, 0.58)	95.7	< 0.001
> 30	3	0.04 (−3.63, 3.71)	93.	< 0.001
NR	2	−3.32 (−9.10, 2.46)	98.8	< 0.001
**Nigella sativa supplementation on BMI**				
Overall	15	−0.51 (−0.85, −0.18)	88.6	< 0.001
*Intervention duration (week)*				
< 12	11	−0.26 (−0.61, 0.10)	82.3	< 0.001
≥ 12	4	−1.23 (−2.05, −0.41)	92.8	< 0.001
*Type of intervention*				
Capsule	7	−0.67 (−1.32, −0.01)	88.9	< 0.001
Oil	8	−0.39 (−0.81, 0.04)	89.9	< 0.001
*Study population*				
NAFLD	4	−0.81 (−1.56, −0.05)	90.6	< 0.001
Obese and Overweight	3	0.03 (−0.49, 0.55)	38.4	0.197
HTN	2	−1.09 (−1.68, −0.51)	66.2	0.086
T2DM	2	−1.52 (−1.88, −1.16)	0.0	0.829
Metabolic Syndrome	3	0.08 (−0.19, 0.35)	39.9	0.189
Hyperlipidemia	1	0.18 (−0.44, 0.80)	—	—
*Sample size*				
≤ 60	4	−0.44 (−1.28, 0.40)	80.0	< 0.001
> 60	11	−0.54 (−0.92, −0.15)	90.6	< 0.001
*BMI*				
≤ 30	12	−0.62 (−0.98, −0.25)	90.5	< 0.001
> 30	3	0.04 (−0.65, 0.74)	44.7	0.164
NR				
*Nigella sativa* supplementation on SBP				
Overall	15	−3.25 (−4.44, −2.06)	74.1	< 0.001
*Age (year)*				
≤ 50	10	−2.31 (−3.63, −0.99)	62.9	< 0.001
> 50	5	−4.63 (−6.27, −2.99)	60.3	0.039
*Intervention duration (week)*				
< 12	14	−3.07 (−4.23, −1.91)	73.4	< 0.001
≥ 12	1	−11.45 (−18.83, −4.07)	—	—
*Type of intervention*				
Capsule	8	−3.25 (−5.26, −1.25)	73.1	< 0.001
Oil	6	−3.53 (−5.15, −1.91)	78.0	< 0.001
*Study population*				
HTN	5	−3.67 (−5.36, −1.98)	81.9	< 0.001
T2DM	1	−9.50 (−14.81, −4.19)	—	—
Metabolic Syndrome	3	−2.33 (−5.57, 0.90)	83.6	< 0.001
Obese and Overweight	3	−2.73 (−8.87, 3.40)	76.9	0.013
Hyperlipidemia	1	−8.18 (−15.62, −0.74)	—	—
NAFLD	2	−2.52 (−4.36, −0.68)	0.0	0.985
*Sample size*				
≤ 60	9	−3.76 (−5.66, −1.86)	79.6	< 0.001
> 60	5	−2.57 (−3.81, −1.33)	45.3	0.104
*BMI*				
≤ 30	11	−3.46 (−4.86, −2.06)	76.7	< 0.001
> 30	2	0.45 (−2.55, 3.45)	0.0	0745
NR	2	−6.63 (−14.17, 0.91)	76.6	0.039
**Nigella sativa supplementation on DBP**				
Overall	15	−2.75 (−4.62, −0.89)	97.9	< 0.001
*Age (year)*				
≤ 50	10	−1.11 (−2.28, 0.05)	85.0	< 0.001
> 50	5	−5.50 (−7.35, −3.66)	91.5	< 0.001
*Intervention duration (week)*				
< 12	14	−2.74 (−4.65, −0.83)	98.1	< 0.001
≥ 12	1	−3.06 (−9.47, 3.35)	—	—
*Type of intervention*				
Capsule	9	−2.30 (−4.10, −0.51)	80.8	< 0.001
Oil	6	−3.16 (−6.40, 0.07)	99.2	< 0.001
*Study population*				
HTN	5	−4.71 (−7.11, −2.30)	96.7	< 0.001
T2DM	1	−5.25 (−8.95, −1.55)	—	—
Metabolic syndrome	3	−2.75 (−5.74, 0.24)	72.4	0.027
Obese and overweight	3	−2.12 (−5.57, 1.34)	92.5	< 0.001
Hyperlipidemia	1	0.38 (−4.59, 5.35)	—	—
NAFLD	2	2.36 (0.67, 4.05)	0.0	0.999
*Sample size*				
≤ 60	9	−3.14 (−5.46, −0.82)	96.2	< 0.001
> 60	6	−1.91 (−3.43, −0.38)	85.5	< 0.001
*BMI*				
≤ 30	11	−2.87 (−4.91, −0.83)	95.9	< 0.001
> 30	2	−1.92 (−5.84, 1.99)	96.1	< 0.001
NR	2	−3.23 (−6.19, −0.28)	0.0	0.952

#### 3.3.2 Effect of *N. sativa* Supplementation on Glycemic Indices

**FIGURE 3:**
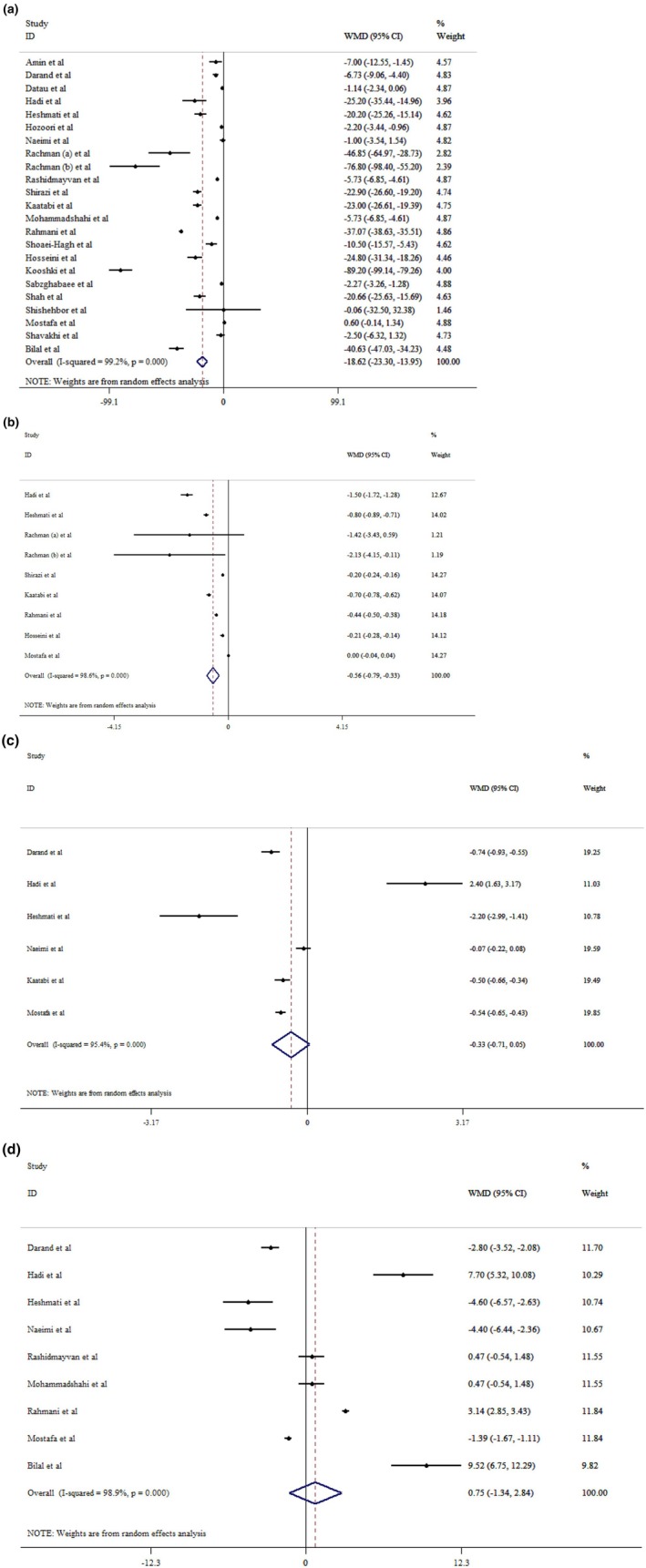
Forest plot detailing mean difference and 95% confidence intervals (CIs) of the effects of *Nigella sativa* supplementation on FBS (a), HbA1c (b), HOMA‐IR (c), and insulin (d) levels.

The results of our analysis indicated that *N. sativa* supplementation substantially decreased FBS (WMD = −18.62 mg/dL; 95% CI: −23.30 to −13.95, *p* < 0.001; *I* ^2^ = 99.2%, *p* < 0.001) (Figure 3a), and HbA1c (WMD = −0.56; 95% CI: −0.79 to −0.33, *p* < 0.001; *I* ^2^ = 98.6%, *p* < 0.001) (Figure 3b), but had no effect on HOMA‐IR (WMD = −0.33; 95% CI: −0.71 to 0.05, *p* = 0.087; *I* ^2^ = 95.4%, *p* < 0.001) (Figure 3c), and insulin (WMD = 0.75 μU/mL; 95% CI: −1.34 to 2.84, *p* = 0.481; *I* ^2^ = 98.9%, *p* < 0.001) levels (Figure 3d). Furthermore, subgroup analysis illustrated that *N. sativa* administration could have pronounced effects across different age groups, treatment duration, and type of supplementation (oil, capsule). However, patients with different health statuses such as metabolic syndrome, T2DM, NAFLD, hyperlipidemia, and HTN benefited from *N. sativa* supplementation substantially (Table 4). Sensitivity analysis showed that HOMA‐IR results changed significantly by removing Hadi et al. [24]. Egger's and Begg's tests showed significant evidence of small‐study effects for FBS (*p* < 0.05). After visually examining the funnel plot, we conducted a trim‐and‐fill analysis due to publication bias (Figure S5). Thus, after adding two imputed studies for FBS, the results showed a still significant change (WMD: −21.14; 95% CI: −28.52 to −13.75, *p* < 0.05).

### 3.4 Effects of *N. sativa* Supplementation on Secondary Outcomes

#### 3.4.1 Effect of *N. sativa* Supplementation on Blood Pressure

**FIGURE 4:**
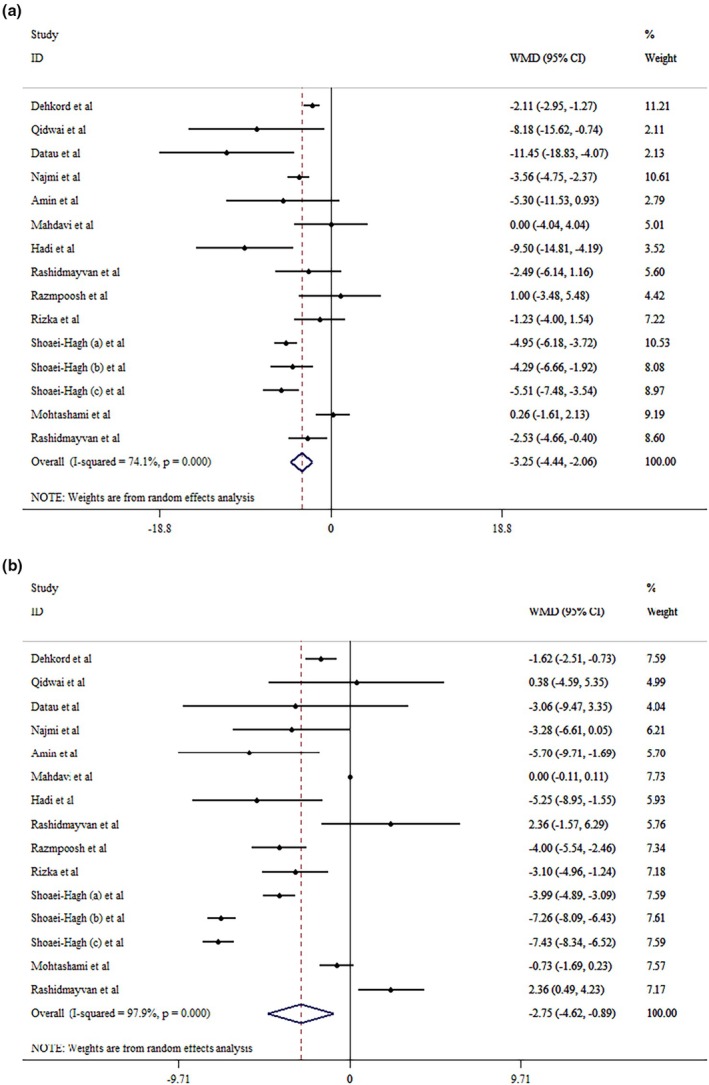
Forest plot detailing mean difference and 95% confidence intervals (CIs) of the effects of *Nigella sativa* supplementation on SBP (a), and DBP (b) levels.

The results of our analysis indicated that *N. sativa* supplementation substantially decreased SBP (WMD = −3.25 mmHg; 95% CI: −4.44, −2.06, *p* < 0.001; *I* ^2^ = 74.1%, *p* < 0.001) (Figure 4a), and DBP (WMD = −2.75 mmHg; 95% CI: −4.62, −0.89, *p* = 0.004; *I* ^2^ = 97.9%, *p* < 0.001) (Figure 4b). Subgroup analysis revealed that *N. sativa* supplementation can improve SBP across all age groups and treatment durations. However, improvements in DBP were observed only in older adults (> 50 years) receiving short‐term supplementation (< 12 weeks). *N. sativa* supplementation was associated with beneficial effects on SBP in patients with non‐alcoholic fatty liver disease (NAFLD), hypertension (HTN), and T2DM who had a lower BMI (< 30). In contrast, significant improvements in DBP were observed in patients with Metabolic syndrome and T2DM (Table 4). Sensitivity analyses confirmed that no individual study notably influenced the overall results. Although Egger's and Begg's tests did not indicate statistically significant small‐study effects for SBP and DBP outcomes (*p* > 0.05), visual inspection of the funnel plots (Figures S3 and S4) revealed potential asymmetry.

## 4 Discussion

To the best of our knowledge this systematic review and meta‐analysis is the first study that has summarized the current available evidence in relation to metabolic effects of *N. sativa* supplementation. Several systematic reviews and meta‐analyses have been conducted in recent years regarding obesity, BP, and glycemic indices [14, 15, 16, 17, 25]. However, the aforementioned studies did not assess certain obesity‐related indices such as HC and WHR. Furthermore, the quality of the evidence was not evaluated using the GRADE approach, which may cast doubt on the reliability of the findings. The current updated meta‐analysis of 31 RCTs suggests that *N. sativa* supplementation may be associated with modest reductions in weight, BMI, SBP, DBP, FBS, and HbA1c, while no significant effects were observed on WC, HC, WHtR, HOMA‐IR, or insulin. Accordingly, there was a high heterogeneity across included studies. To this end, subgroup analysis based on age groups, intervention duration, type of *N. sativa* , study population, sample size, and BMI of the participants was conducted to investigate the source of this variability.

Although several outcomes in our meta‐analysis showed statistically significant changes, the magnitude of these effects was modest and may limit their clinical relevance. Reductions in BW (about 2 kg), BMI (about 0.5 kg/m^2^), and systolic/diastolic blood pressure (about 2–3 mmHg) observed with *N. sativa* supplementation are relatively small, particularly given that most included trials were of short duration (4–24 weeks). In clinical practice, more substantial weight loss (≥ 5% of baseline body weight) and decrease in blood pressure (5 mmHg) are generally considered a threshold for meaningful improvements in cardiometabolic risk factors [26, 27]. Consequently, the modest absolute changes seen in our analyses may represent small effects rather than definitive therapeutic benefits in isolation. Such modest reductions could contribute to cardiometabolic risk improvement when combined with diet, physical activity, or other interventions, but they are unlikely to independently yield large clinical benefit in real‐world settings.

High heterogeneity was observed across most outcomes (*I* ^*2*^ ranging from 74.1% to 99.5%). Subgroup analyses indicated that differences in study population and baseline BMI partially explained this variability, with greater effects on FBS, weight, and BMI observed in specific populations (e.g., patients with T2DM or metabolic syndrome) and in those with BMI ≤ 30. Other factors, such as intervention duration or supplementation form, did not substantially reduce heterogeneity, suggesting that baseline characteristics of participants are the main contributors to variability in the effects of *N. sativa* supplementation. Given the high variability and modest absolute effect sizes, these findings should be interpreted with caution.

The finding that *N. sativa* supplementation was associated with modest reductions in weight and BMI suggests a potential role in supporting weight management, although the absolute changes were small and observed over short‐term interventions. These findings may be attributed to the bioactive components present in *N. sativa* , particularly thymoquinone, which has been shown to exert anti‐inflammatory, antioxidant, and metabolic modulatory effects [28, 29, 30]. In addition, it seems that *N. sativa* may be involved in the adipogenesis and fat oxidation pathways to affect body composition [31]. Moreover, thymol, another bioactive component of *N. sativa* seed, is a lipase inhibitor and a reducing agent of unsaturated fatty acids, which causes weight loss in adults [32]. While these favourable properties of *N. sativa* was not enough to have a significant effect on other anthropometric indices too. However, subgroup analysis revealed clinically relevant findings which indicated that *N. sativa* was capable of improving weight and BMI levels in long‐term (> 12 weeks) supplementation. This finding suggests that sustained supplementation may be necessary to achieve meaningful anthropometric improvements. Also, it has been demonstrated that *N. sativa* supplementation exerts the most pronounced benefits in patients with T2DM.

In addition, *N. sativa* supplementation was associated with modest reductions in SBP and DBP. *N. sativa* supplementation improved SBP across all age groups and treatment durations. However, improvement in DBP was observed only in older adults (> 50 years) receiving short‐term supplementation (< 12 weeks). The findings suggest that *N. sativa* exerts time‐dependent effects on different clinical outcomes and this contrast may reflect differences in the physiological mechanisms underlying these outcomes. The antioxidant capacity of *N. sativa* , which can be related to the presence of thymoquinone, flavonoids, nigericin, trans‐anethole, and limonene, increases endothelial function contributing to reduced BP [33, 34]. In addition, the blood pressure‐lowering benefits of *N. sativa* are also attributed to its polyphenol concentration [35]. Also, flavonoids have been shown to exert an inhibitory effect on the angiotensin‐converting enzyme (ACE), which may improve normal endothelial function and reduce BP [36, 37].

Furthermore, the findings showed that *N. sativa* supplementation was associated with modest reductions in FBS and HbA1c, suggesting a potential supportive role in glycemic management, although the absolute changes were small and observed over short‐term interventions. However, observed publication bias suggests that the magnitude of the effect of *N. sativa* on FBS should be interpreted with caution. These beneficial effects of *N. sativa* were observed across all age groups, various supplementation durations, and different forms of *N. sativa* (e.g., oil, capsule) in patients with metabolic syndrome, T2DM, NAFLD, hyperlipidemia, HTN to improve FBS level. Mechanistically, these findings may be attributed to thymoquinone, which has been shown to improve insulin sensitivity, improve β‐cell function, reduce oxidative stress, and suppress inflammatory pathways involved in glucose metabolism [23, 38].

Ten studies had higher quality with a lower risk of bias. An assessment of the quality of evidence using the GRADE approach indicated that the quality of evidence for weight, BMI, SBP was moderate, all of which showed statistically significant improvements with *N. sativa* supplementation. This suggests a reasonably high level of confidence in the observed effects for these outcomes, though future studies could further strengthen the evidence base. In contrast, the quality of evidence for HC and WHR was rated as high, despite the fact that *N. sativa* supplementation did not produce statistically significant changes in these measures.

The strengths of this study include the subgroup analysis based on intervention duration, which enables clinicians to determine the safe length of *N. sativa* administration with greater confidence. In addition, the study was conducted in accordance with current methodological standards, including the GRADE approach. Nevertheless, some of the outcomes were rated as moderate for quality assessment. Although the moderate quality of evidence indicates a likely effect, that it warrants cautious interpretation of findings. Also, many of the studies we included had limited sample sizes and varied in terms of patient characteristics and study design, potentially impacting the outcomes. Additionally, different types of *N. sativa* were used in the studies we reviewed, and it was impossible to isolate their individual effects due to limitations in the current research. In addition, the results of publication bias and the sensitivity analysis suggest that the findings of weight, WC, WHtR, FBS, and HOMA‐IR should be interpreted with caution.

## 5 Conclusion

This systematic review and meta‐analysis suggests that *N. sativa* supplementation is associated with modest improvements in weight, BMI, blood pressure, FBS, and HbA1c, particularly in interventions lasting longer than 12 weeks. However, the evidence was inconsistent or of low certainty for several cardiometabolic outcomes, and no significant effects were observed for markers of insulin resistance or central adiposity. Therefore, these findings should be interpreted cautiously, and further high‐quality, long‐term randomized controlled trials are required to clarify the magnitude and clinical relevance of these effects.

## Author Contributions

**Mohsen Khaleghian:** project administration, supervision, writing – review and editing, visualization, writing – original draft, funding acquisition, investigation. **Mahsa Mahmoudinezhad:** writing – original draft, funding acquisition, writing – review and editing, visualization, validation, formal analysis, supervision, resources. **Amir Hossein Faghfouri:** writing – original draft, validation, formal analysis, project administration, visualization, writing – review and editing, resources. **Vali Musazadeh:** conceptualization, investigation, methodology, project administration, formal analysis, resources, supervision, writing – review and editing, visualization. **Farshad Teymoori:** writing – original draft, funding acquisition, visualization, formal analysis. **Ali Arash Anoushirvani:** investigation, funding acquisition, writing – original draft, validation, formal analysis, resources. **Melika Jahangir:** conceptualization, investigation, validation, software, project administration, supervision, writing – review and editing, funding acquisition, writing – original draft.

## Funding

The authors have nothing to report.

## Ethics Statement

The authors have nothing to report.

## Consent

The authors have nothing to report.

## Conflicts of Interest

The authors declare no conflicts of interest.

## Supporting information


**Figure S1:** Funnel plot of the effect of *nigella sativa* on weight.
**Figure S2:** Funnel plot of the effect of *Nigella sativa* on BMI.
**Figure S3:** Funnel plot of the effect of *Nigella sativa* on SBP.
**Figure S4:** Funnel plot of the effect of *Nigella sativa* on DBP.
**Figure S5:** Funnel plot of the effect of *Nigella sativa* on FBS.
**Table S1:** The search strategy using MESH terms and keywords.

## Data Availability

The data that support the findings of this study are available from the corresponding author upon reasonable request.
