# Supplementary material for: The Effect of Nigella sativa Supplementation on Cardiometabolic Health in Patients With Metabolic Diseases: A GRADE‐Assessed Systematic Review and Meta‐Analysis
Source: Endocrinol Diabetes Metab. 2026 Mar 20;9(2):e70207. doi: 10.1002/edm2.70207 (PMC13093553; doi:10.1002/edm2.70207)
Supplement: Supplementary file 1 — Figure S1: Funnel plot of the effect of nigella sativa on weight. Figure S2: Funnel plot of the effect of Nigella sativa on BMI. Figure S3: Funnel plot of the effect of Nigella sativa on SBP. Figure S4: Funnel plot of the effect of Nigella sativa on DBP. Figure S5: Funnel plot of the effect of Nigella sativa on FBS. Table S1: The search strategy using MESH terms and keywords. [file EDM2-9-e70207-s001.docx]

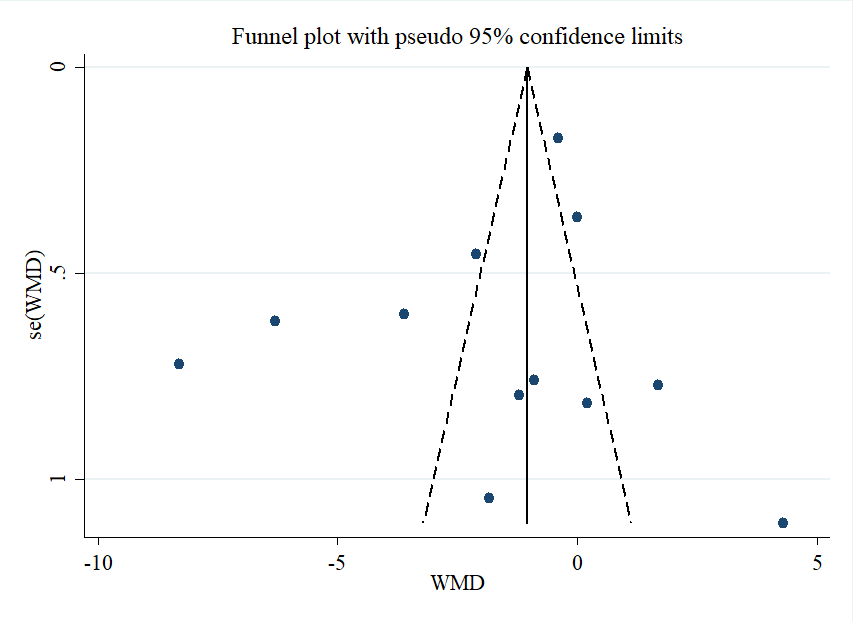


**Supplementary Figure 1.** Funnel plot of the effect of nigella sativa on weight.


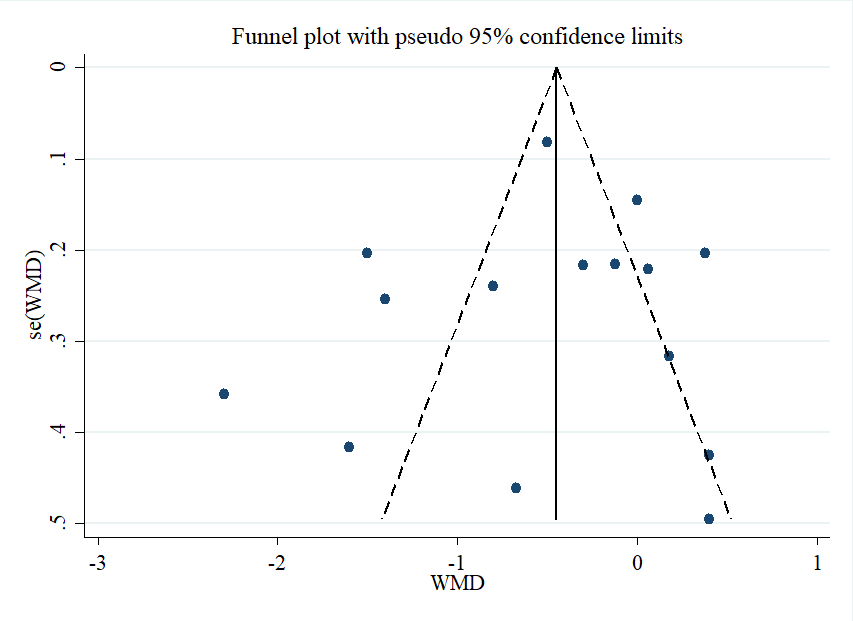


**Supplementary Figure 2.** Funnel plot of the effect of nigella sativa on BMI.


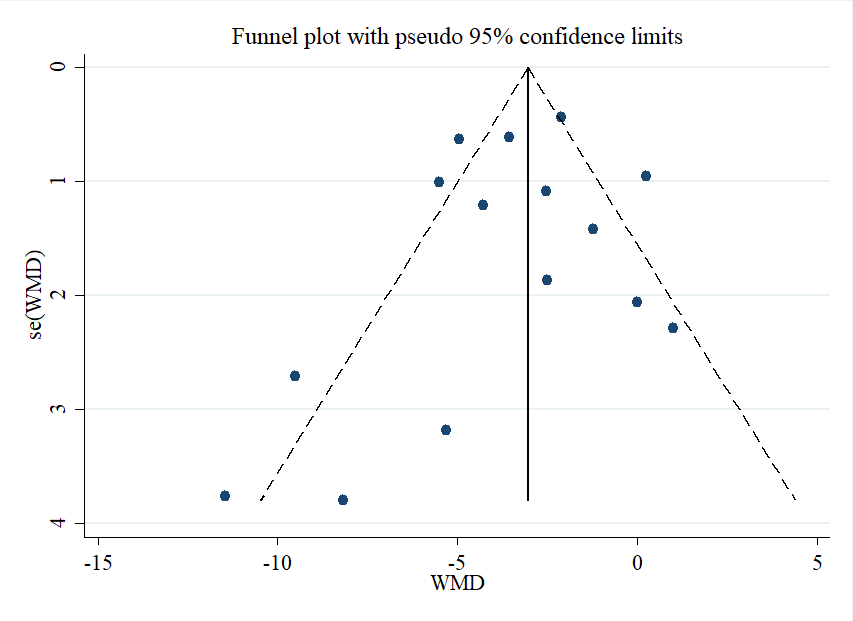


**Supplementary Figure 3.** Funnel plot of the effect of nigella sativa on SBP.


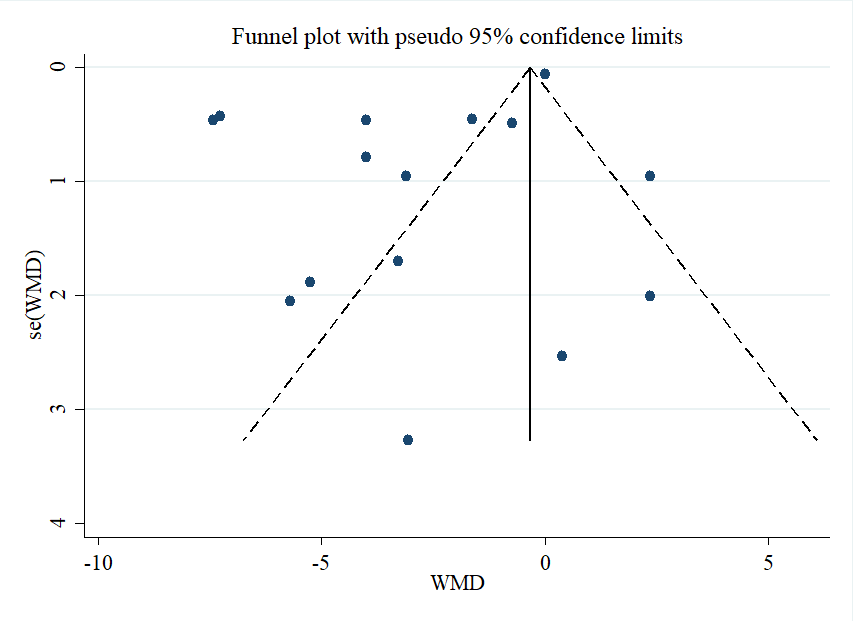


**Supplementary Figure 4.** Funnel plot of the effect of nigella sativa on DBP.


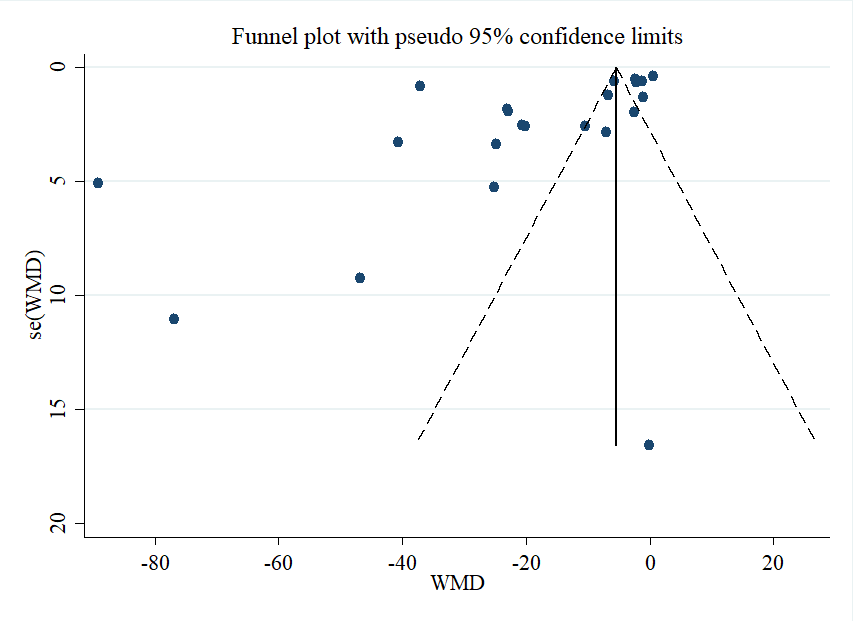


**Supplementary Figure 5.** Funnel plot of the effect of nigella sativa on FBS.

**Supplementary Table 1**. The search strategy using MESH terms and keywords.

| **Keywords were defined using Medical Subject Headings (MeSH) using for databases** |
| --- |
| (“*Nigella sativa*”[ Title/Abstract] OR "*Nigella sativa*"[Mesh] OR “*Nigella sativa* "[All Fields] OR “Cuminum”[ Title/Abstract] OR “black cumin*”[Title/Abstract] OR “black caraway”[Title/Abstract] OR “thymoquinone”[ Title/Abstract] OR “TQ” OR “kalonji”[ Title/Abstract] OR “Black seed”[Title/Abstract] OR “Black seed”[All Fields]) AND "body weight" [Title/Abstract] OR "body weight changes" [Title/Abstract] OR "body mass index" [Title/Abstract] OR "weight loss" [Title/Abstract] OR "obesity" [Title/Abstract] OR "body weight" [Title/Abstract] OR "body mass index" [Title/Abstract] OR "BMI" [Title/Abstract] OR "waist circumference" [Title/Abstract] OR "WC" [Title/Abstract] OR "hip circumference"[Title/Abstract] OR "HC" [Title/Abstract] OR "waist-to-hip ratio"[Title/Abstract] OR "WHR"[Title/Abstract] AND ("randomized controlled trial"[Publication Type] OR "controlled clinical trial"[Publication Type] OR "controlled clinical trial"[All Fields] OR randomized[Title/Abstract] OR randomized [Title/Abstract] OR placebo[Title/Abstract] OR "clinical trials as topic"[MeSH Terms] OR "cross-over studies"[MeSH Terms] OR "cross-over studies"[All Fields] OR "cross over studies"[All Fields] OR "Cross-over study"[All Fields] OR "Cross over study"[All Fields] OR "clinical trial"[Publication Type] NOT animals[All Fields]). |
